# The Work Already Done in the Direction of Standardizing Fluid Preparations

**Published:** 1890-02

**Authors:** 


### THE WORK ALREADY DONE IN THE DIRECTION OF STANDARDIZING FLUID PREPARATIONS.

The first and most notable advance made in the direction of supplying standardized preparations not open to the dangers of the existing Pharmacopœial processes for fluid extracts, was by Messrs. Parke, Davis & Co., who introduced in 1883, a class of assayed preparations which were entitled Normal Liquids. The standard decided upon for these fluids was the result of long experience in the collection, purchase, examination and analysis of crude drugs with a determination of the amount and character of their active principles. The reliability of normal liquids soon led to their large consumption, and the medical profession have evinced their preference for them to such an extent as to make them now an established and popular method of exhibiting the toxic and narcotic drugs.

Normal liquids may be defined to be concentrated tinctures, the methods of manufacture of which serve as models for imitions. They represent more closely than fluid extracts made by the present Pharmacopœial methods the average standard strength crude drugs. The simplest explanation of their nature would probably be to regard them as fluid extracts adjusted by assay to a fixed standard of strength which make them absolutely uniform in composition and therapeutic action.

The favor with which normal liquids, and assayed products generally, have been received by representative men of the medical profession, has led us to believe that the best interests of pharmacy will not be served unless these or like preparations are officially recognized. For concentrated tinctures of a definite strength, the name "normal liquids" appears to be happily chosen, as it implies a definite standard of strength. The list should embrace preparations of the more potent crude drugs, 1 Ccm. representing 1 gramme of drug of standard strength.

It does not seem to us from a careful review of all efforts made in this direction that any have met with equal acceptance, or merit as much appreciation. Whatever may prove to be the decision of the Committee as to making such assayed preparations official, there can never be any question as to whom the honor of their actual practical introduction is due.

As the time approaches when the revision is to take place (and in the minds of thinking men the standardization of fluid extracts is now an accepted fact), there will no doubt be many competitors for this honor who may claim by reason of a mushroom-like growth in the field of this new departure, official recognition for scientific work.

It will be necessary on the part of the Committee of Revision, therefore, to carefully investigate the claims in this direction, and when awarding the credit for such work to see that they do not place the laurels upon the wrong brow.

Unsupported and disinterested scientific labor, no matter from what source, should always be welcomed with the endorsement of scientific men, and we sincerely trust that the efforts made in this direction by those deserving it, will receive full appreciation at the hands of the compilers of the forthcoming Pharmacopœia.—Medical Age.

